# Treatment of Palm Oil Refinery Effluent Using Tannin as a Polymeric Coagulant: Isotherm, Kinetics, and Thermodynamics Analyses

**DOI:** 10.3390/polym12102353

**Published:** 2020-10-14

**Authors:** Nik Mohd Farid Mat Yasin, Md. Sohrab Hossain, Abdul Khalil H.P.S., Muzafar Zulkifli, Adel Al-Gheethi, Ahmad Jaril Asis, Ahmad Naim Ahmad Yahaya

**Affiliations:** 1 Malaysian Institute of Chemical and Bioengineering Technology (MICET), Universiti Kuala Lumpur, Alor Gajah 78000, Melaka, Malaysia; nik.farid@s.unikl.edu.my (N.M.F.M.Y.); muzafar@unikl.edu.my (M.Z.); 2 Sime Darby Research Sdn Bhd, Carey Island 42900, Selangor, Malaysia; ahmad.jaril.asis@simedarbyplantation.com; 3 School of Industrial Technology, Universiti Sains Malaysia (USM), Penang 11800, Malaysia; akhalilhps@gmail.com; 4 Micropollutant Research Centre (MPRC), Faculty of Civil Engineering & Built Environment, Universiti Tun Hussein Onn Malaysia, Parit Raja 86400, Johor, Malaysia; adel@uthm.edu.my

**Keywords:** polymeric coagulant, industrial effluent treatment, isotherm modeling, thermodynamics, kinetics

## Abstract

The refining of the crude palm oil (CPO) generates the palm oil refinery effluent (PORE). The presence of high contents of biochemical oxygen demand (BOD), chemical oxygen demand (COD), turbidity, and suspended solids (SS) in PORE encourages the determination of an effective treatment process to minimize the environmental pollution and preserve aquatic life. In the present study, a biodegradable natural polymer, namely tannin, was utilized as a coagulant to treat PORE. The coagulation experiment was conducted using a jar test apparatus. The tannin coagulation efficiency was evaluated based on the BOD, COD, turbidity, and SS removal from PORE by varying the tannin dose (50–300 mg/L), pH (pH 4–10), treatment time (15–90 min), and sedimentation time (15–90 min). It was found that the maximum removal of BOD, COD, turbidity, and SS was 97.62%, 88.89%, 93.01%, and 90.21%, respectively, at pH 6, a tannin dose of 200 mg/L, 60 min of coagulation time, and 60 min of sedimentation time. Analyses of isotherm models revealed that the Freundlich isotherm model was well fitted with the coagulation study. Kinetics studies show that the pseudo-second-order kinetics model was the well-fitted kinetics model for the BOD, COD, turbidity, and SS removal from PORE using tannin as a polymeric coagulant. The determination of thermodynamics parameters analyses revealed that BOD, COD, turbidity, and SS removal from PORE was spontaneous, exothermic, and chemical in nature. The finding of the present study shows that tannin as a natural polymeric coagulant would be utilized in PORE treatment to avoid toxic sludge generation.

## 1. Introduction

Palm oil is the most used vegetable oil and agricultural product worldwide. The demand to produce palm oil is increasing, owing to its multipurpose application in both food and non-food products [1]. At present, palm oil is used as a viable feedstock to produce biodiesel due to its lower price and elevated oil yield compared with other edible oils [2]. The worldwide production of palm oil is reported to be over 70 million metric tons in 2018, and it is expected that palm oil production will surplus over 75 million metric tons by 2020 [3]. Malaysia is the second-largest palm oil producer and exporter country. The oil palm cultivation and the production of palm oil have contributed significantly to the Malaysian national economic growth. However, the production of palm oil is producing a huge quantity of solid waste and industrial effluents in the forms of empty fruit bunch (EFB), mesocarp fiber, palm kernel shell, palm oil refineries effluent (PORE), and palm oil mill effluent (POME) [1,4]. Among the palm oil industry effluent, PORE contains approximately 94–96% water, 4–6% suspended organic particles, and 0.6–0.7% oil [5,6]. Although the organic pollutant concentration in PORE is much lower than POME, the concentration of biochemical oxygen demand (BOD), chemical oxygen demand (COD), turbidity and suspended solids (SS) in PORE is higher than the industrial effluents discharge limits set by the Department of Environment (DoE), Malaysia [7]. Therefore, it is necessary to treat PORE to minimize the residual BOD, COD, turbidity and SS concentration below the recommended discharge limits for industrial effluents set by DoE, Malaysia.

The most common treatment technology for treating PORE is the biological treatments using an anaerobic or aerobic ponding process, because of its low operating cost [6]. The biological ponding process normally requires pre-treatment to remove residual oil and to minimize the concentration of the organic pollutant by dissolved air flotation (DAF) and coagulation using alum as a coagulant, respectively [6,8,9]. However, the biological treatment is a lengthy process, which requires prolonged treatment time for the degradation of organic particles [10]. Additionally, a lack of regulatory control and release of greenhouse gases from the biological treatment process are the major drawbacks of the existing PORE treatment process. The biological wastewater treatment process is viewed as the second-largest greenhouse gases producer in Malaysia. Gases produced from the biological treatment process are corrosive and odorous because of containing hydrogen sulfide and ammonia [8]. Although the Malaysian palm oil industries have employed advance biological treatment processes with the capability of capturing biogas from the biological ponding process, it requires careful monitoring as the pollutant degrades by the microorganisms [8,11]. In addition, the residual BOD, COD, turbidity, and SS concentrations in treated PORE do not comply with the standard discharge limits for industrial effluents set by DoE, Malaysia. Thus, it urges post-treatment to minimize the residual BOD, COD, turbidity, and SS concentration below the recommended discharge level set by DoE, Malaysia.

The coagulation is the most preferred technology for the industrial effluent treatment due to its well-known capability of aggregating and destabilizing colloidal and suspended particles [11,12]. This technology has been extensively utilized to treat various industrial effluents, including PORE, using various chemical-based commercial coagulants, such as alum, aluminum chloride, poly-aluminum chloride, and poly ferric sulfate. The distinct advantages of this process are the short treatment time, effective removal of both inorganic and inorganic pollutants, easy handling, and availability [11,13]. The major shortcoming of the coagulation process is the generation of hazardous sludge, which requires a costly disposal method. The sludge generated in the coagulation process contains toxic metals ion due to the utilized alum, aluminum chloride, aluminum or based polymeric salts including poly-aluminum chloride (PAlCl), and polyferric sulfate as a coagulant [11,13]. Therefore, scientists are looking for natural polymer as an alternative to commercial coagulant for applications in environmental technologies, wherein tannin as a natural polymeric coagulant would be highly promising. Tannin is an anionic natural polymer, which is extracted from the secondary metabolites in plant matrices [14]. It is a water-soluble polyphenolic compound with a molecular weight from 500 Dalton to some thousand Dalton. Tannin could be utilized as a bio-coagulant due to the presence of carboxyl and hydroxyl groups. Anionic and cationic tannins were used as a coagulant to treat wastewater in an environmentally friendly manner [14,15,16]. The studies reported that tannin has the potential to replace the commercial coagulant due to its compatible coagulation efficiency with a commercial coagulant. As a natural polymer, tannin does not contain any metal ion in its structure. In addition, tannin is a natural biodegradable polymer, and therefore, the generated sludge after coagulation could be utilized as organic fertilizer in oil palm plantation [14,15]. In addition, the irregular shape of tannin provides higher affinity to suspended and colloidal particles, which enhance the flocculation [16].

In the coagulation process, the negatively charged organic particles agglomerate with positively charged coagulant due to the ionization and adsorption of organic particles on the surface of the coagulant. However, the correlation between the adsorbate and adsorbent is homogeneous or heterogeneous with uniform or non-uniform distribution [11]. Therefore, the determination coagulation using tannin as a coagulant for the BOD, COD, SS, and turbidity removal is important to observe a reasonable correlation between the adsorbate and coagulant during coagulation. Generally, isotherm mathematical modeling explains the adsorbate–coagulant amalgamation. In addition, the study of kinetics and thermodynamics modeling describes coagulation behavior. Thus, it bears considerable interest on the determination tannin coagulation behavior using equilibrium modeling, kinetics, and thermodynamics modeling on BOD, COD, turbidity, and SS removal from PORE. The main objective of the present study is to determine the tannin coagulation efficiency as an effective and alternative coagulant for PORE treatment. Therefore, in the present study, the influence of tannin as a polymeric coagulant was determined on the removal of BOD, COD, turbidity, and SS removal from PORE with varying tannin doses, pH, treatment time, and sedimentation time. In addition, isothermal, kinetics, and thermodynamics models were employed to evaluate the tannin coagulation behavior. The finding of the present study will be useful to determine an environmentally friendly coagulant for the PORE treatment.

## 2. Materials and Methods

### 2.1. Sample Collection and Preparation

Raw palm oil refinery wastewater was obtained from the palm oil refinery, Sime Darby Plantation, Carey Island, Selangor, Malaysia. Polyethene containers were used to collect the PORE. Subsequently, the collected PORE was cooled at ambient temperature before being stored at 4 °C. The tannin used as a coagulant in the present study was supplied by Qwatso Chemical Sdn Bhd, Sabah, Malaysia. Based on the company information, it is a chemical modified cationic polymer of tannin extract obtained from the Black wattle (*Acacia mearnsii*). The cationic tannin extract was obtained in a dark brown color powder form. 

### 2.2. Physicochemical Analyses of PORE

The physical properties of PORE, including pH and temperature in treated and untreated PORE, were determined using a pH meter equipped with a temperature sensor (Mettler Toledo F20). Dilute sulfuric acid (1M) and sodium hydroxide (1M) solutions were utilized to adjust the desired pH of the solutions. The BOD concentration in untreated and treated PORE was determined by using the HACH respirometric method (HACH method 10099). Ten mL of untreated or treated PORE were taken into the BOD track sample bottle and filled with deionized water. Subsequently, a BOD nutrient buffer pillow and lithium hydroxide powder pillow were taken into the BOD reagent bottle and stirred before incubating into a BOD track incubator at 20 °C for five days. The determination of BOD, COD, turbidity, and SS concentration in treated and untreated PORE were conducted by following the standard methods for water and wastewater analyses [17]. The COD concentration in untreated and treated PORE was conducted with the reactor digestion method (HACH method, 8000) by using a HACH DR 2800 spectrometer. Two mL of homogenized PORE and 2 mL of deionized water were taken into high range (HR) COD digestion vials (range 20 to 1500 mg/L) for a sample and blank test, respectively. Then, the COD digestion vials were taken into the COD reactor and heated at 150 °C for 2 h. Later, the COD digestion vials were cooled to 120 °C, and the COD (mg/L) was measured using a DRB 200 reactor. The SS concentration in untreated and treated PORE was evaluated by employing the photometric method (HACH method 8006). A sample cell containing 10 mL of blended untreated and treated PORE was taken in the sample cell holder, and the SS concentration in untreated and treated PORE was determined using a spectrophotometer (HACH DR 2800). The turbidity analyses in untreated and treated PORE were determined using a Hach 2100Q Portable Turbidimeter. Five mL of PORE was placed in a clean container and filled with deionized water to 15 mL, and the sample was replaced into the cell. A thin film of silicone oil was added and then wiped with a soft cloth. Then, the sample cell was taken into the cell compartment, and the lid was closed to analyses.

### 2.3. Coagulations Experiments Procedure

**Figure 1:**
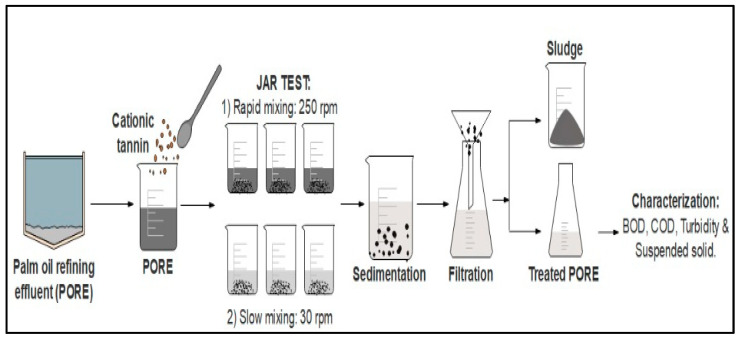
Schematic diagram for the tannin coagulation experimental procedure.

The tannin coagulation efficiency for BOD, COD, turbidity, and SS removal from PORE was determined using a jar test apparatus with tannin as a natural coagulant. The jar test apparatus utilized for the coagulation experiments consists of six beakers with six paddle rotors to stir. The experiment was carried out by taking 500 mL of untreated PORE at ambient temperature (28 ± 1 °C) with varying pH (4, 5, 6, 7, 8, 9, and 10), tannin doses (50, 100, 150, 200, 250, and 300 mg/L), a rapid mixing time of 3 min at 250 rpm, coagulation times (slow mixing) (15, 30, 45, 60, 75, and 90 min) at 30 rpm to allow flocculation and sedimentation time (15, 30, 45, 60, 75, and 90 min). The residual COD, BOD, turbidity, and SS concentrations in the treated PORE were evaluated using the following equation:

(1) $$\mathrm{Removal}\;(\%)=\left(1-\frac{C_{t}}{C_{i}}\right)\times 100$$

where *C_i_* is the BOD, COD, turbidity, and SS concentrations in untreated PORE and *C_t_* is the BOD, COD, turbidity, and SS concentrations in treated PORE at time *t*. The experiments were carried out in triplicate, and the data were represented as the mean value ± standard deviation from the triplicate experiment runs. The schematic diagram for the tannin coagulation experimental procedure is shown in Figure 1.

### 2.4. Coagulation Isotherm Modeling

The coagulation adsorbent–adsorbate amalgamation was determined using Freundlich and Langmuir isotherms model equations for BOD, COD, turbidity, and SS removal from PORE using tannin as a polymeric coagulant. The experiments were carried out with changing doses from 50 to 300 mg/L at pH 6.0, 3 min rapid mixing time, 60 min slow mixing time, and 60 min sedimentation time. The suitability of the applied isotherm model equations was determined by the use of linear regression method with relating the coefficient of determination (R^2^) to the experimental data. The coagulation at equilibrium (*q_e_*) for the removal of BOD, COD, turbidity, and SS from PORE using tannin as a coagulant was computed using Equation (2):

(2) $$q_{e}=\frac{C_{i}-C_{e}}{D}\times V$$

where *q_e_* denotes the coagulation at equilibrium (mg/mg); *V* refers to the volume (mL) of PORE, and *D* refers to the doses of tannin as (mg). The liner form of the Freundlich isotherm model equation can be expressed as presented in Equation (3):

(3) $$\log q_{e}=\log K_{f}+\frac{1}{n}\log C_{e}$$

where *K_f_* refers to the Freundlich affinity coefficient (L/mg), and *n* is the Freundlich exponential constant. The linear form of the Langmuir isotherm model equation can be expressed as below:

(4) $$\frac{1}{q_{e}}=\frac{1}{abC_{e}}+\frac{1}{b}$$

where *a* indicates the Langmuir constant and *b* indicates the highest coagulation value of tannin for BOD, COD, turbidity, and SS removal from PORE.

### 2.5. Kinetics Modeling

The prime importance of the coagulation kinetics is to elucidate the physical and chemical behavior of adsorbent (physisorption or chemisorption) as well as to determine the adsorbate uptake rate by adsorbent [18]. In the present study, pseudo-first-order and pseudo-second-order kinetic model equations were employed to evaluate the kinetics behavior for BOD, COD, turbidity, and SS removal from PORE using tannin as a polymeric coagulant. Wherein, the coagulation experiments were carried out with varying coagulation temperature from 30 to 80 °C as a function of slow mixing time from 5 to 90 min with a constant pH of 6.0, a constant rapid mixing time of 3 min, and a sedimentation time of 60 min. The pseudo-first-order kinetic model equation can be written as below [13]:

(5) $$\ln(q_{e}-q_{t})=\ln q_{e}-k_{1}t$$

where *q_e_* represents the BOD, COD, turbidity, and SS (mg/mg) removal from PORE at equilibrium; and *q_t_* signifies the BOD, COD, turbidity, and SS (mg/mg) removal from PORE at time *t* (min). *k*_1_ signifies the pseudo-first-order coagulation rate constant (min^−1^). The pseudo-second-order equation can be expressed as presented in Equation (6):

(6) $$\frac{t}{q_{t}}=\frac{1}{k_{2}q_{e}^{2}}+\frac{t}{q_{e}}$$

where *k_2_* refers to the pseudo-second-order coagulation rate constant (mg/mg/min).

### 2.6. Thermodynamics Analysis

The thermodynamics parameters, for instance, Gibbs free energy (∆*G*^0^), enthalpy changes (∆*H*^0^), and entropy change (∆*S*^0^), were analyzed to determine the thermodynamics behavior of the tannin coagulation for BOD, COD, turbidity, and SS removal from PORE. The coagulation experiments were carried out with varying coagulation temperatures (30, 40, 50, and 80 °C) at pH 6.0, a rapid mixing time of 3 min, a slow mixing time from 5 to 90 min, and a 60 min sedimentation time. The thermodynamics properties were analyzed by using the following equations:

(7) $$\Delta G^{0}=-RT\ln K_{0}$$

(8) $$\Delta G^{0}=\Delta H^{0}-T\Delta S^{0}$$

(9) $$\ln K_{0}=\frac{\Delta S^{0}}{R}-\frac{\Delta H^{0}}{RT}$$

where *R* indicates the universal gas constant (8.314 × 10^−3^ kJ mol^−1^ K^−1^), *T* is the Kelvin temperature, and *K_o_* is the Gibbs constant. The *K_o_* values were calculated from the ratio of *q_e_* to *C_e_*. ∆*H*^0^ and ∆*S*^0^ were calculated from the slope and intercept from the linear plot of ln*K_o_* versus *1*/*T*, respectively.

## 3. Results and Discussion

### 3.1. Physicochemical Characterization of PORE

The physicochemical properties of untreated PORE were determined, as shown in Table 1. It was observed that pH (5.86 ± 0.22) and temperature (28 ± 2 °C) in untreated PORE were within the industrial effluents discharge limits set by DoE, Malaysia (Table 1). However, the BOD (518 ± 5 mg/L), COD (1350 ± 25 mg/L), turbidity (465 ± 8 mg/L), and SS (1430 mg/L) concentrations were higher than the industrial effluents discharge limits set by DoE, Malaysia, under the 5th regulations for industrial effluent disposal [7]. The higher BOD, COD, turbidity, and SS concentrations in untreated PORE reveal that the PORE must be treated to minimize the residual pollutant below discharge limits. This is because the untreated or partially treated PORE discharge in a watercourse may pose detrimental effects to the environment and aquatic life. In addition, the treatment methods chosen for PORE treatment must be effective and ecofriendly without generating secondary waste such as hazardous sludge. Similarly, Hassan et al. [19] determined that the BOD and COD concentration in untreated PORE were 2600 and 3000 mg/L, respectively. The variation of the pollutant concentration in PORE might due to the utilization of the refining process applied. The PORE collected in the present study was from the Sime Darby palm oil refinery, wherein it is utilized as a chemical refining process to produce fresh palm oil from CPO. In addition, the efficient monitoring on utilizing chemicals in the palm oil production and refining process by the Research and Development (R&D), Sime Darby Research Sdn Bhd toward the palm oil sustainability would affect the lower pollutant concentration in the PORE. 

### 3.2. Treatment of PORE Using Tannin as a Coagulant

**Figure 2:**
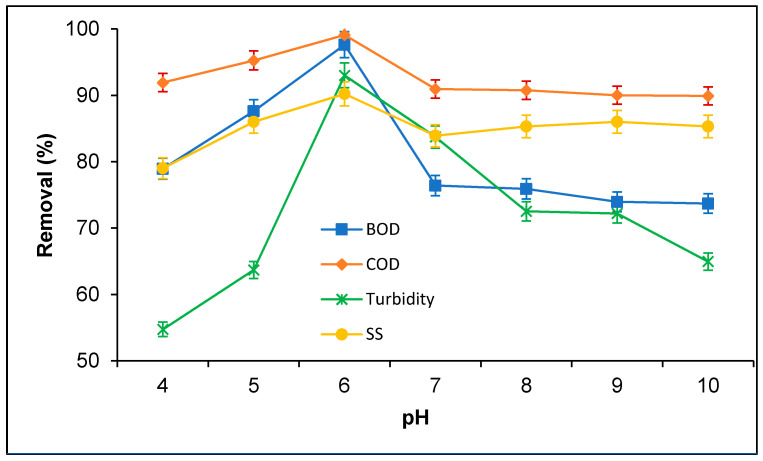
Effect of pH on the removal of BOD, COD, turbidity and SS from PORE using tannin as a coagulant. Experimental condition: doses 200 mg/L, coagulation time 60 min, and sedimentation time 60 min.

pH potentially influenced the coagulation efficiency from the industrial effluents [10]. The influences of pH on BOD, COD, turbidity, and SS removal from PORE using tannin as a polymeric coagulant were evaluated, as present in Figure 2. It was observed that BOD, COD, and SS removal increased with increasing pH from pH 4.0 to 6.0; then, it decreased gradually until pH 7.0, and thereafter, the decrease of BOD, COD, turbidity, and SS removal from PORE was negligible with the further increase of pH from pH 7.0 to 10.0. In the case of turbidity, the BOD, COD, turbidity, and SS removal percentage from PORE was increased with increasing pH up to pH 6.0 and gradually decreased with further increasing the pH. The maximum BOD, COD, turbidity, and SS removal from PORE obtained were 97.62%, 94.67%, 93.01%, and 90.22% respectively, at pH 6.0. The possible mechanisms of tannin coagulation of PORE could be attributed to the interaction between the coagulant and adsorbate particles. At lower pH, some of the suspended organic particles, particularly the organic acids present in the PORE, might not interact with the coagulant particles and therefore lower the BOD, COD, turbidity, and SS removal. However, the increase of pH in the aqueous solution increases the cationic charge on the surface of the tannin, which substantially increased the affinity of tannin to bind suspended organic particles and hence increased coagulation efficiency [20]. At over pH 6.0, the decrease of the tannin coagulation efficiency with enhanced pH might be due to the competition between OH^−^ and organic particles to attach with tannin. At higher alkaline regions, more OH^−^ attached with tannin, which turns to the catatonic tannin less positive charge and diminishes the attraction to the anionic organic particles present in the PORE, and therefore, it decreased the removal efficiency of BOD, COD, turbidity, and SS from PORE [17]. Dela Justina et al. [21] observed that the tannin coagulation efficiency for the removal of color, turbidity, and total solids from dairy wastewater was influenced by pH and reached a maximum pH over 5.0. Graham et al. [22] reported on the decrease of the tannin coagulation efficiency due to the losses of cationic charge and aqueous solubility with pH. Tannin has a strong aqueous solubility below pH. Over the pH, the solubility of tannin decreases with increasing pH, which may diminish the binding of the suspended and organic particles and therefore decrease the removal of BOD, COD, turbidity, and SS removal from PORE [22,23].

pH influences the prominence of the most chemical reactions in the aquatic environment [17]. When the pH values are higher and lower than neutral pH in treated effluent, it will affect the receiving water quality and its aquatic life. In the present study, the highest BOD, COD, turbidity, and SS removal was gained at pH 6.0, which is closed to the neutral pH value and within the permissible pH value range set by DoE, Malaysia. Thus, the treated PORE could be discharged into surface water without interfering with the surface water quality and aquatic life. In addition, the pH value of the raw PORE is 5.86 ± 0.22, which is close to the optimal pH, 6.0, for the maximum removal of BOD, COD, TSS, and turbidity from PORE. Thus, the treatment PORE using tannin as a natural coagulant would not require any pH adjustment, which substantially minimizes the PORE treatment cost.

**Figure 3:**
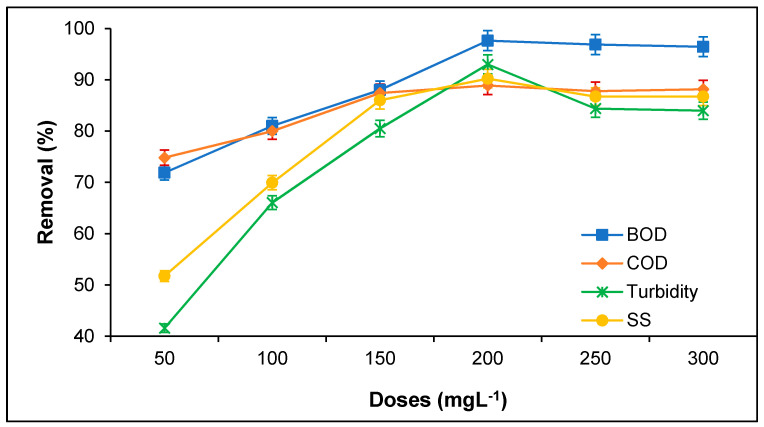
Effect of tannin doses on the removal of BOD, COD, turbidity, and SS from PORE using tannin as a coagulant. Experimental condition: pH 6.0, coagulation time 60 min, and sedimentation time 60 min.

The influence of tannin doses on BOD, COD, turbidity, and SS removal from PORE was determined with varying tannin doses from 50 to 300 mg/L, as presented in Figure 3. It was observed that the tannin doses potentially influence the coagulation efficiency of PORE. The minimal BOD, COD, turbidity, and SS removal from PORE was found at tannin doses of 50 mg/L. However, the BOD, COD, turbidity, and SS removal percentage enhanced with increasing tannin doses up to 200 mg/L; thereafter, the BOD, COD, turbidity, and SS removal from PORE diminished with the further increasing tannin doses. The highest BOD, COD, turbidity, and SS removal obtained were 97.62%, 88.89%, 93.01%, and 90.21%, respectively.

BOD, COD, turbidity, and SS removal increased with increasing tannin doses due to the increase of cationic charged in the PORE to attract negative charge organic particles for floc formation [12]. Ahead of the optimal coagulant doses (over 200 mg/L), BOD, COD, turbidity, and SS removal decreased due to the reversal charge that took place on the surface of the tannin with the surplus amount of tannin doses [11,19]. Due to the reversible charge on tannin with the excessive amount of tannin doses, it has minimized the affinity of hydrogen bonding with the suspended organic particles, and hence, it decreases the removal of BOD, COD, turbidity, and SS [11,22]. Similarly, Hossain et al. [11] observed that the coagulation efficiency on the removal of BOD, COD, and SS increased with increasing coagulant doses up to 1 g/L and decreased with further increasing coagulation doses. Lopes et al. [16] utilized a tannin-based bio-coagulant for the decolorization of synthetic effluent and found the complete decolorization with 180 mg/L tannin doses. Beltrán-Heredia and Sánchez-Martín [20] studied the removal of turbidity from municipal wastewater using tannin as a bio-coagulant. The study reported that the tannin doses potentially influenced the turbidity removal efficiency and obtained complete turbidity removal at 100 mg/L tannin doses. In the present study, the removal of BOD, COD, turbidity, and SS were obtained about 98%, 89%, 93%, and 90%, respectively with 200 mg/L tannin doses. The variation of results obtained in the present study might occur due to the different types of wastewater and a higher concentration of suspended organic particles. 

**Figure 4:**
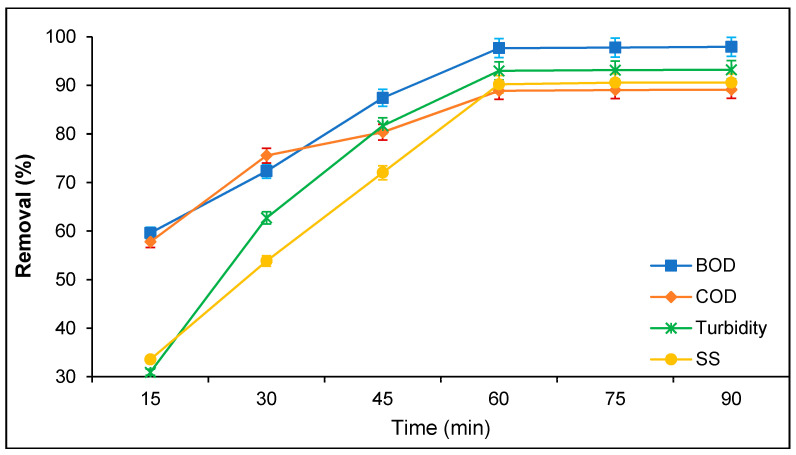
Effect of treatment time on the removal of BOD, COD, turbidity, and SS from PORE using tannin as a coagulant. Experimental condition: pH 6.0, doses 200 mg/L, and sedimentation time 60 min.

BOD, COD, turbidity, and SS removal from PORE using tannin as a coagulant was determined with varying treatment time from 15 to 90 min, as shown in Figure 4. It was found that the treatment time expressively influences tannin coagulation efficiency for BOD, COD, turbidity, and SS removal from PORE. The BOD, COD, turbidity, and SS removal from PORE increased with increasing treatment time from 15 to 60 min; thereafter, the BOD, COD, turbidity, and SS removal was negligible. At 15 min treatment time, the BOD, COD, turbidity, and SS removal were 59.60%, 57.78%, 30.93%, and 33.57%, respectively. However, BOD, COD, turbidity, and SS removal enhanced to 97.66%, 88.89%, 90.21%, and 93.01%, respectively, at 60 min treatment time. The treatment time is important for a coagulation process to form between the coagulant and organic particles present in the wastewater. Inefficient floc formation during PORE treatment may cause collisions between the tannin particles and suspended organic particles present in PORE. After rapid mixing, the organic particles present in the PORE require time and slow mixing to induce with tannin particles for increasingly forming larger agglomerates to settle down [16,24]. The minimal BOD, COD, turbidity, and SS removal obtained at 15 min treatment time might be due to the low collisions between suspended organic particles and tannin particles. With increasing slow mixing time, the collisions between suspended organic particles and tannin particles were also increased, and therefore, they increased the BOD, COD, turbidity, and SS removal from PORE. However, the increase of BOD, COD, turbidity, and SS removal from PORE was gained over 60 min treatment time due to the saturation of the surface of tannin organic particles. The weak collision between saturated tannin particles and suspended organic particles diminish the BOD, COD, turbidity, and SS removal efficiency from PORE. A similar observation has reported by several studies for the removal of suspended organic particles from various wastewater using natural coagulants [16,24,25].

**Figure 5:**
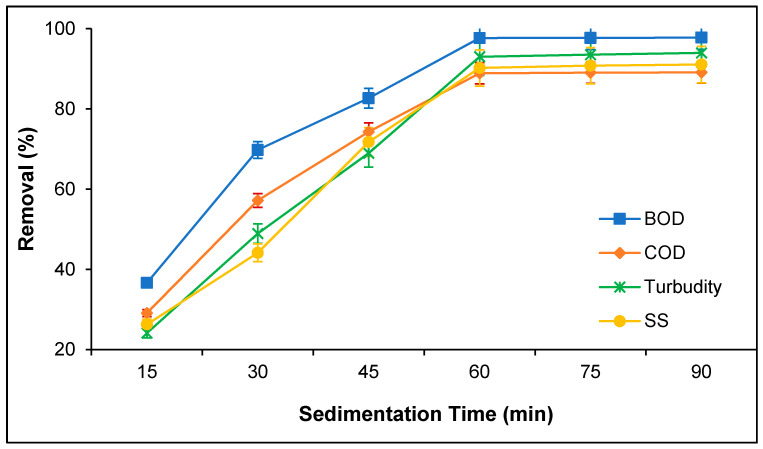
Effect of sedimentation on the removal of BOD, COD, turbidity, and SS from PORE using tannin as a coagulant. Experimental condition: pH 6.0, doses 200 mg/L, and coagulation time 60 min.

Sedimentation time potentially influences the coagulation efficiency for the removal of suspended and colloidal organic particles from industrial effluents [11,24]. Sedimentation time is crucial in the coagulation and flocculation process for the agglomeration and floc formation of the suspended organic particles with the coagulant particles to settle down. The effect of the sedimentation time for BOD, COD, turbidity, and SS removal from PORE using tannin as a polymeric coagulant was determined, as presented in Figure 5. It was found that BOD, COD, turbidity, and SS removal enhanced with sedimentation time and reached the stationary phase over 60 min sedimentation time. Zahrim et al. [26] reported that the coagulation is a necessary process, which aggregates colloidal and suspended organic particles to larger flocs with the surface neutralization of coagulant particles. However, the degree of agglomeration depends on the sedimentation time. To effectively agglomerate the suspended organic particles present in PORE, it requires a certain duration of sedimentation time to form floc by entrapping the suspended organic particles with tannin and settling down the floc into the bottom of the aqueous phase [27]. Based on the findings of the present study, it can be postulated that the sufficient sedimentation time is over 60 min for BOD, COD, turbidity, and SS removal from PORE using tannin as a coagulant. 

Table 2 shows the residual BOD, COD, SS, and turbidity concentration in treated PORE using tannin as a coagulant at the optimal experimental condition of pH.6.0, tannin doses of 200 mg/L, coagulation time of 60 min, and sedimentation time of 60 min. It was found that the BOD (12 ± 5 mg/L) and COD (150 ± 5 mg/L) concentrations are below the recommended standard B discharge limits set by DoE, Malaysia (Table 1). However, the SS concentration (140 ± 5 mg/L) is still higher than the industrial effluents discharge limits (50–100 mg/L). There are no discharge limits for the turbidity in industrial effluents that have been set by DoE, Malaysia. Dela Justina et al. [21] observed that tannin has the comparative coagulation efficiency of polyaluminum chloride for the removal of color, turbidity, and total solids from dairy wastewater. The findings of the present study reveal that tannin has the potential to be an alternative to commercial inorganic coagulant for treating PORE. However, it requires further study to minimize the residual SS concentration below the recommended discharge limits to discharge treated PORE to a watercourse safely.

### 3.3. Isotherm Modeling for PORE Treatment Using Tannin as a Polymeric Coagulant

**Figure 6:**
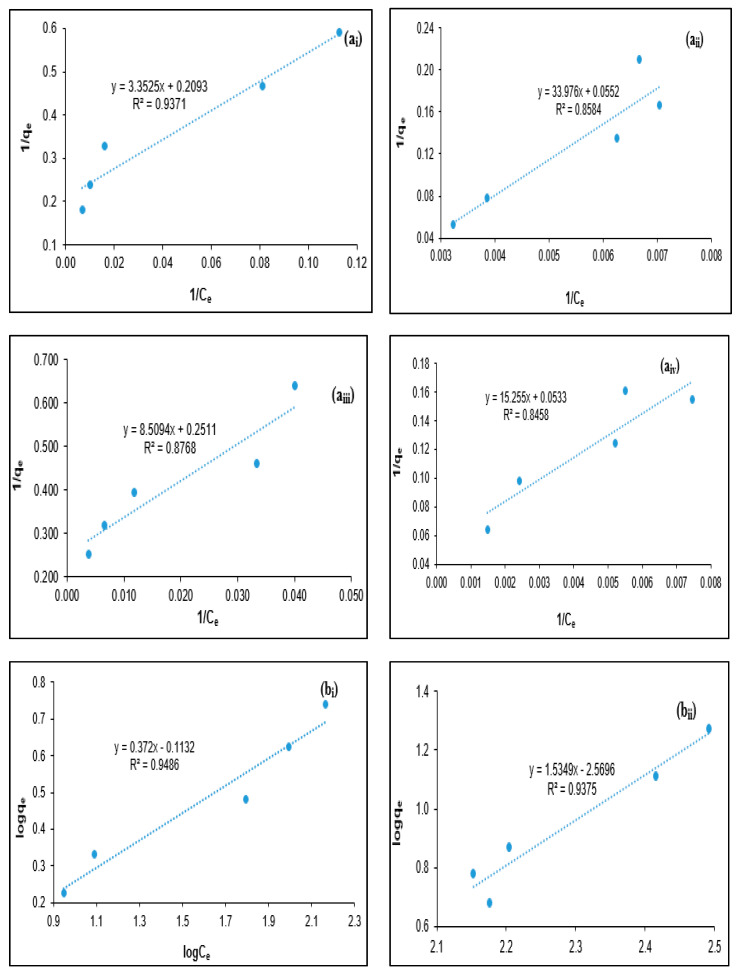

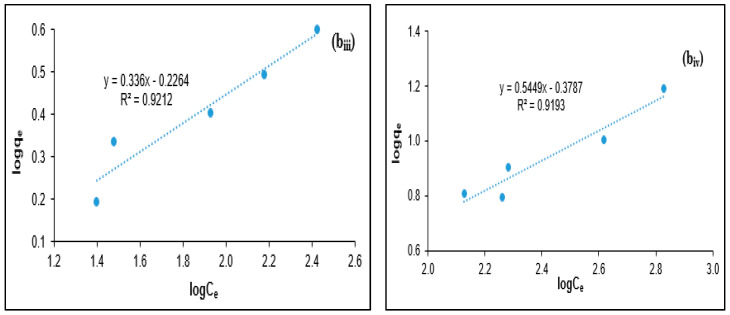
Langmuir (**a**) and Freundlich (**b**) adsorption isotherm model for the removal of (**i**) BOD (**ii**) COD (**iii**) turbidity and (**iv**) SS from PORE using tannin as a bio-coagulant.

The isotherm modeling studies express the dependency of the colloidal and suspended organic particles with coagulant during the coagulation process. In addition, the coagulation isotherm modeling equations are effective in explaining coagulation behavior. Among these isothermal models, the Freundlich and Langmuir isothermal models are the most utilized isotherm model models to determine the nature of the coagulation process [13,24,26]. In the present study, Langmuir and Freundlich isotherm models were utilized to express the coagulation behavior of tannins on BOD, COD turbidity, and SS removal from PORE, as shown in Figure 6.

The Langmuir isotherm model expresses that the coagulation adsorption process occurs in a homogeneous distribution and monolayer formation [17,27]. The Langmuir isotherms for BOD, COD, turbidity, and SS removal from PORE using tannin as a natural polymeric coagulant are presented in Figure 6a. The Langmuir constant values (a, L/mg) and the maximum coagulation (b, mg/mg) values were calculated from Equation (4) using the slop and intercept data obtained from Figure 5. It was found that the calculated Langmuir constant (a) values were 0.0624, 0.0016, 0.0295, and 0.0035 L/mg for BOD, COD, turbidity, and SS removal from PORE, respectively. Wherein, the maximum adsorption (b) values were calculated to be 4.7778, 18.1160, 3.9825, and 18.7612 mg/mg for BOD, COD, turbidity, and SS removal from POR, respectively. However, the calculated *a* (L/mg) and *b* (mg/mg) values were positive, suggesting that the BOD, COD, turbidity, and SS removal from PORE using tannin as a coagulant is possible.

The Freundlich isotherm model expresses that the coagulation adsorption ensues on the surface of coagulant particles in a heterogeneous distribution with non-uniform distribution and multilayer formation [16,28]. However, the Freundlich isotherm model describes that the adsorption process is reversible, and therefore, it does not restrict to the monolayer formation [11,28]. Figure 6b shows the Freundlich isotherms for BOD, COD, turbidity, and SS removal from PORE using tannin as a polymeric coagulant. The Freundlich affinity constant (*K_f_*) values were determined to be 0.7705, 0.0027, 0.5937, and 0.4181 L/mg for the BOD, COD, turbidity, and SS removal from PORE, respectively. In addition, the Freundlich exponential constant (*n*) values were determined to be 2.6882, 0.6515, 2.9762, and 1.8352 for BOD, COD, turbidity, and SS removal from PORE; those are within the range of 1 to 10. Thus, it can be postulated that the BOD, COD, turbidity, and SS removal from PORE using tannin as a coagulant is doable [28].

Table 3 shows the coefficient of determination (R^2^) values for Langmuir and Freundlich isotherms on BOD, COD, turbidity, and SS removal from PORE, subjected to tannin as a coagulant. The R^2^ values of the Langmuir isotherm model were 0.9371, 0.8584, 0.8768, and 0.8458 for BOD, COD, turbidity, and SS removal, respectively. Wherein, the R^2^ values of the Freundlich isotherm model were found to be 0.9486, 0.9375, 0.9212, and 0.9193 for BOD, COD, turbidity, and SS removal from PORE, respectively. It was observed that R^2^ values for Langmuir and Freundlich isotherm models were higher than 0.80, suggesting that both Freundlich and Langmuir isotherm models were fit with the experimental data to designate the coagulation behavior for BOD, COD, turbidity, and SS removal from PORE using tannin as a coagulant. However, the greater R^2^ values (>0.91) of the Freundlich isotherm model imply the better fit with experimental data for BOD, COD, turbidity, and SS removal from PORE using tannin as a coagulant. This indicates that the BOD, COD, turbidity, and SS removal from PORE were conducted on the heterogeneous surface of tannin particles with multilayer formation and non-uniform distribution. In addition, the tannin coagulation process for the removal of BOD, COD, turbidity, and SS from PORE is reversible, and therefore, the tannin coagulation process does restrict to the monolayer formation. Similarly, Badawi et al. [29] reported that the Freundlich isotherm model was well fitted with experimental data for the removal of aluminum and lead from wastewater using modified chitosan–tannin acid as an adsorbent. Alijerf [30] reported that the Freundlich isotherm model was the best-fitted model equation to describe the adsorption behavior for the elimination of heavy metals from tannery industrial effluent using modified zeolite as an adsorbent.

### 3.4. Kinetic Study

**Figure 7:**
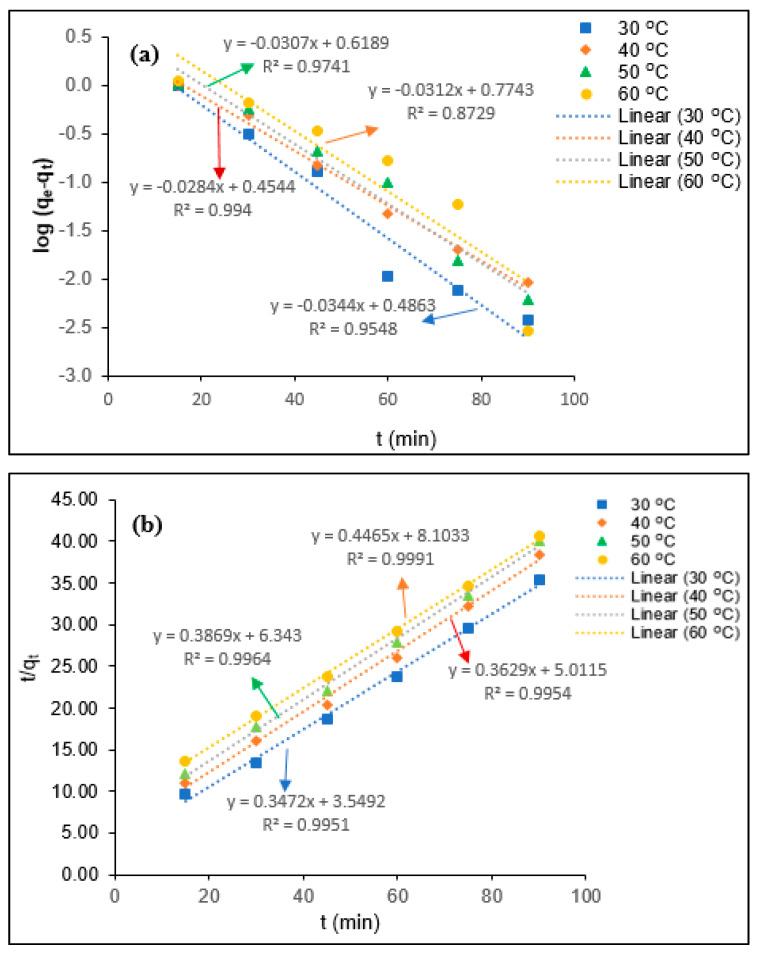
Kinetics modeling for the removal of BOD from PORE using tannin as a coagulant. (**a**) Pseudo-first-order kinetics model, (**b**) Pseudo-second-order kinetics model.

**Figure 8:**
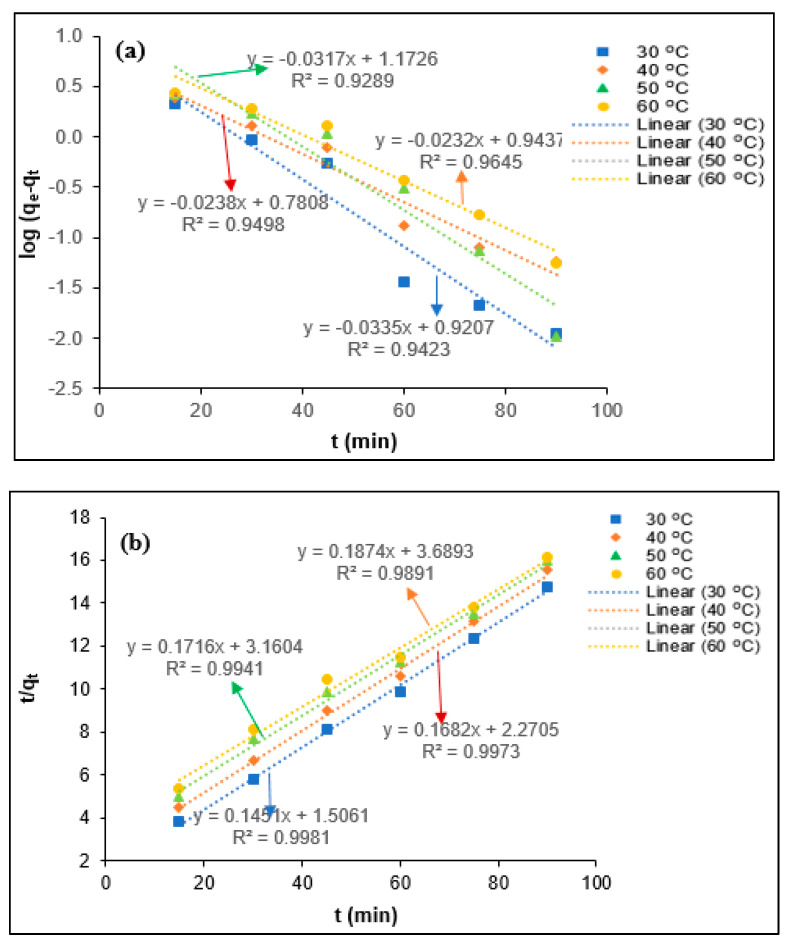
Kinetics modeling for the removal COD from PORE using tannin as a coagulant. (**a**) Pseudo-first-order kinetics model, (**b**) Pseudo-second-order kinetics model.

**Figure 9:**
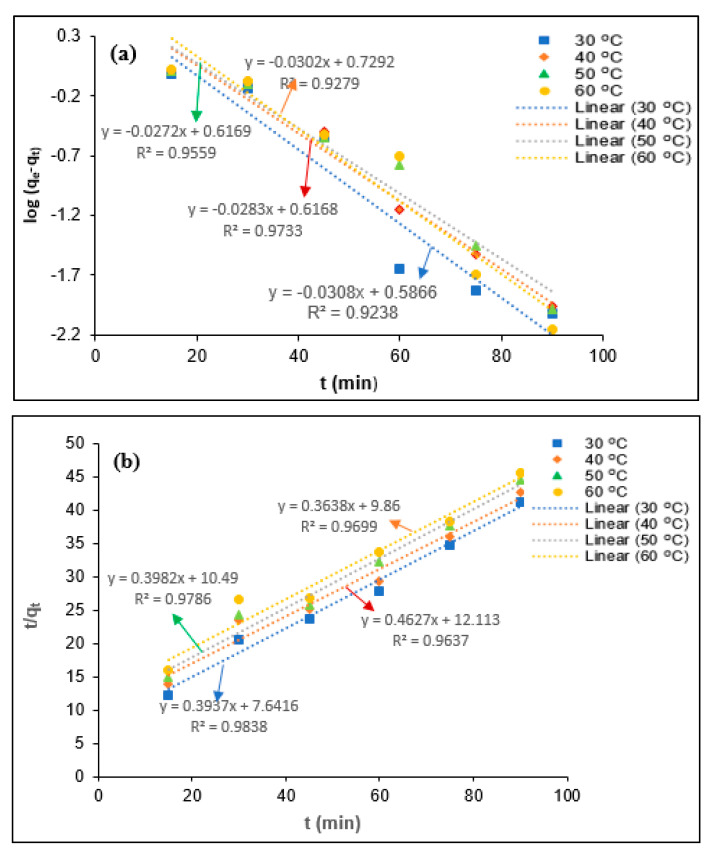
Kinetics modeling for the removal turbidity from PORE using tannin as a coagulant. (**a**) Pseudo-first-order kinetics model, (**b**) Pseudo-second-order kinetics model.

**Figure 10:**
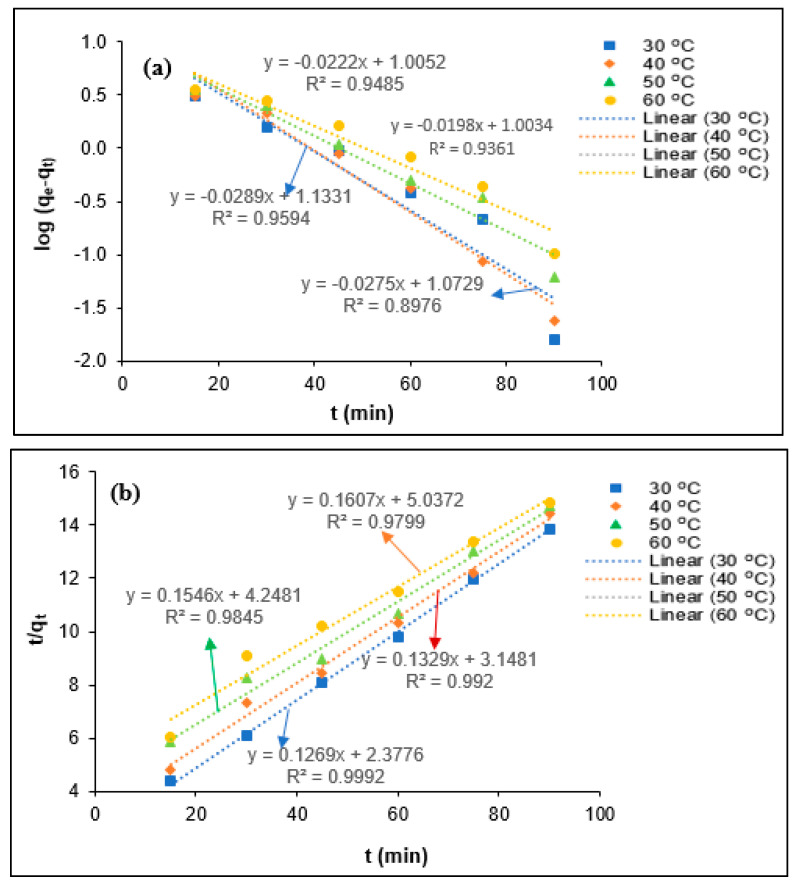
Kinetics modeling for the removal SS from PORE using tannin as a coagulant. (**a**) Pseudo-first-order kinetics model, (**b**) Pseudo-second-order kinetics model.

The determination of the coagulation kinetics is important to predict the coagulation behavior of the colloidal and suspended organic particles present in the industrial effluent. Generally, the coagulation mechanisms for the elimination of the colloidal and suspended organic particles depend on the chemical characteristics of coagulant and mass transfer phenomena [13,31]. The coagulation kinetics describes the removal efficiency of the colloidal and suspended organic particles on the surface of the coagulant at each equilibrium contact time. To determine the tannin coagulation mechanisms for BOD, COD, turbidity, and SS removal PORE, the pseudo-first-order (a) and pseudo-second-order (b) kinetic models were utilized in the present study, as shown in Figure 7, Figure 8, Figure 9 and Figure 10, respectively. The uptake rate for BOD, COD, turbidity, and SS removal was evaluated by fitting the experimental data with the pseudo-first-order kinetic model and pseudo-second-order kinetic model equations. Wherein, the values of the first-order rate constant (k_1_) and *q_e_* (mg/mg) were calculated using data obtained from the slope and intercept of the plot ln (*q_e_*-*q_t_*) against *t* (min). The pseudo-second-order rate constant (k_2_) and *q_e_* (mg/mg) were calculated using the data obtained from the intercept 1/k_2_ *q_e_*^2^ and the slope 1/*q_e_* of the plot t/qt against *t* (min).

**Figure 11:**
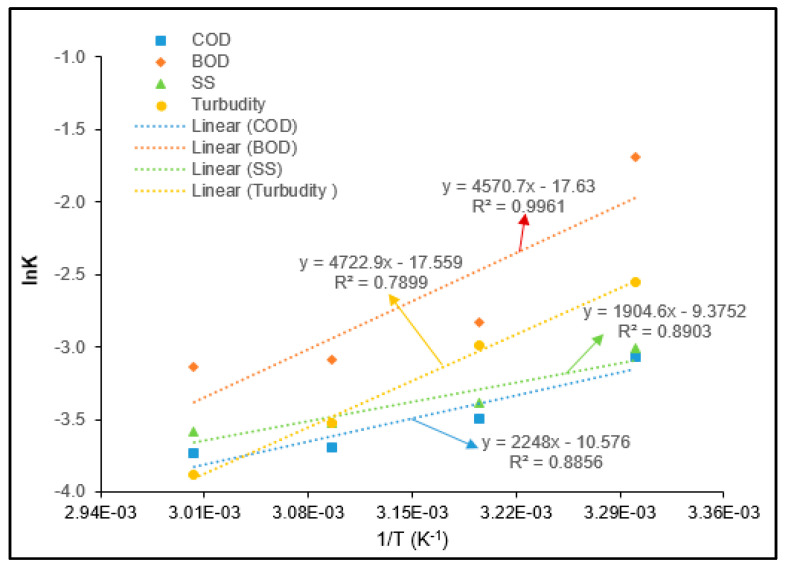
Thermodynamics modeling for the removal of BOD, COD, turbidity, and SS from PORE using tannin as a coagulant.

The kinetics parameters such as *q_e_* (experimental and calculated), R^2^, k_1_, and k_2_ values were determined from Figure 8, Figure 9, Figure 10 and Figure 11 for BOD, COD, turbidity, and SS removal from PORE using tannin as a coagulant, and they are presented in Table 4. It was found that *q_e_* (experimental) values slightly reduced with the elevated temperature from 30 to 60 °C. The decrease of *q_e_* values with the elevated temperature might be attributed to the swelling effect of the chemically modified tannin particles and increase in the kinetics movement of adsorbate with increasing temperature, which allows the adsorbate to escape from the surface of tannin particles; hence, the adsorption efficiency slightly decreased. In addition, the pseudo-first-order rate constant (*k*_1_) was found to decrease with increasing temperature from 30 to 60 °C, and it increases with further increasing temperature from 40 to 60 °C for the removal of BOD from PORE using tannin as a coagulant. The decrease of the coagulation rate with increasing temperature attributes to the exothermic nature of the natural polymer. With the further increase of the temperature, the viscosity of the PORE decreases, which enhances the collision between the adsorbate and modified tannin particles and therefore increases the kinetics rate for the BOD removal. However, the influence of temperature on the pseudo-first-order rate constant (*k*_1_) was found to be inconsistent for the removal of COD, turbidity, and SS. In the case of the pseudo-second-order rate constant (*k*_2_), the *k*_2_ values decreased with increasing temperature from 30 to 50 °C and increased with further increasing temperature for BOD, COD, and turbidity removal from PORE. Wherein, the *k*_2_ values decreased with increasing temperature from 30 to 60 °C for the removal of SS from PORE using tannin as a coagulant.

In the present study, the best-fitted kinetics model was determined based on the R^2^ values and the difference between actual *q_e_* values and theoretical *q_e_* values. As can be seen in Table 3, the R^2^ values for the pseudo-second-order kinetic model were closer to unity than the pseudo-first-order kinetic model. Moreover, it was found that the actual *q_e_* values are more closely matched with the calculated *q_e_* values from the pseudo-second-order kinetic model than those from the pseudo-first-order kinetic model. Thus, it can be concluded that the pseudo-second-order kinetic model is the best-fitted kinetics model to elucidate the coagulation mechanisms for BOD, COD, turbidity, and SS removal from PORE using tannin as a coagulant. Similarly, Badawi et al. [29] reported that the pseudo-second-order kinetics model was best fitted with experimental data for the removal of aluminum and lead from wastewater using modified chitosan–tannin acid as a coagulant.

### 3.5. Adsorption Thermodynamics 

The thermodynamic behavior for BOD, COD, turbidity, and SS removal from PORE using tannin as a coagulant was determined with varying temperature from 30 to 60 °C. The thermodynamics parameters such as Gibbs free energy changes (∆*G*^0^), enthalpy changes (∆*H*^0^), and entropy changes (∆*S*^0^) were determined from the Van’t Hoff plot, as given in Figure 11.

Table 5 shows the ∆*G*^0^, ∆*H*^0^, and ∆*S*^0^ values for BOD, COD, turbidity, and SS removal from PORE by using tannin as a coagulant. It was found that the ∆G^0^ values were negative for BOD, COD, turbidity, and SS removal from PORE. This reveals that the coagulation process was spontaneous and favorable. The spontaneity of the tannin coagulation process for BOD, COD, turbidity, and SS removal from PORE can be further illuminated with the decrease of the Gibbs free energy values with increasing temperature. It found that the ∆*G*^0^ decreased with increasing temperature from 30 to 80 °C, which indicates that the external energy source influences the tannin coagulation efficiency. The obtained ∆*H*^0^ values were determined to be −39.267, −18.690, −15.838, and −38.003 kJ/mol for BOD, COD, turbidity, and SS removal from PORE, respectively. These negative ∆*H*^0^ values reveal that the tannin coagulation process for BOD, COD, turbidity, and SS removal from PORE was exothermic. The positive values of ∆*S*^0^ indicate an increase of the entropy of the solid and liquid interface with the increase of the temperature. However, the negative ∆*H*^0^ value (∆*H*^0^ < 1) and the positive ∆*S*^0^ value (∆*S*^0^ > 1) reveal that the BOD, COD, turbidity, and SS removal from PORE using tannin as a coagulant was spontaneous at all studied temperatures.

Generally, coagulation adsorption is a physical process, and the adsorption process is the exothermic reaction when used natural fiber as an adsorbent. However, the presence of various reactive functional groups (i.e., –COOH, –OH, NH_2_^+^) on the surface of the adsorbent might involve an adsorption process with binding suspended and colloidal organic particles on the surface of the adsorbent [26]. Therefore, the coagulation adsorption process for the removal of suspended organic particles from industrial effluents can be best described by the physicochemical process. Wherein, the suspended organic particles adsorb on the surface physically (physisorption) by the weak Vander Walls Force, and the suspended organic particles bind on the surface by the active functional groups present on the surface or form new chemicals by an ion exchange reaction [11,18]. The ∆*G*^0^ values are the indicator to determine the physisorption or chemisorption process. The ∆*G*^0^ values for the physisorption process are within the range from −20 to 0 kJ/mole, wherein the ∆*G*^0^ values for the chemisorption process are within the range from −80 to −400 kg/mole [32]. In the present study, the ∆*G*^0^ values for BOD, COD, turbidity, and SS removal from PORE using tannin as a coagulant were −83 to −88 kJ/mole, −45 to −48 kJ/mole, −82 to −87 kJ/mole, and −39 to 42 kJ/mole, respectively. This indicates that the ∆*G*^0^ values for the removal of BOD and turbidity were within that range of the chemisorption process, wherein the ∆*G*^0^ values for COD and SS removal are between the range of the physisorption and chemisorption process.

## 4. Conclusions

In the present study, the influence of tannin as a polymeric coagulant was determined on BOD, COD, turbidity, and SS removal from PORE. It was found that the tannin coagulation efficiency was potentially influenced by pH, tannin doses, coagulation times, and sedimentation time. The highest BOD, COD, turbidity, and SS removal from PORE using tannin as a coagulant were 97.62%, 88.89%, 93.01%, and 90.21% respectively at pH 6.0, tannin doses of 200 mg/L, a coagulation time of 60 min, and a sedimentation time of 60 min. The Freundlich isotherm model was a well-fitted isotherm model for BOD, COD, turbidity, and SS from PORE using tannin as a coagulant. The best-fitted kinetics model was the pseudo-second-order kinetic model to elucidate the coagulation mechanisms. The thermodynamics parameters analyses revealed that the tannin coagulation process for BOD, COD, turbidity, and SS from PORE was spontaneous and exothermic. The findings of the present study reveal that tannin has the potential to utilize in PORE treatment to minimize the residual BOD, COD, turbidity, and SS concentration below the stringent industrial effluent discharge limits set by DoE, Malaysia.

## Figures and Tables

**Table 1:** Physicochemical properties of untreated palm oil refinery effluent (PORE) and their respective discharge limits set by DoE Malaysia in the 5th industrial effluent regulation in 2009 [7].

Parameters	Unit	Concentration	* Discharge Limits [6]	
			Standard A	Standard B
pH	-	5.86 ± 0.22	6.0–9.0	5.5–9.0
Temperature	°C	28 ± 2	≤40	≤40
BOD	mg/L	518 ± 5	20	40
COD	mg/L	1350 ± 25	80	200
SS	mg/L	1430 ± 12	50	100
** Turbidity	mg/L	464 ± 8	-	-

* Standard A is applicable to discharges into any inland waters within catchment. areas listed in the Third Schedule, while Standard B is applicable to any other. inland waters or Malaysian waters. ** No discharge limits set by DoE.

**Table 2:** Residual pollutants concentration in treated PORE using tannin as a coagulant.

Parameters	Unit	Concentration	Residual Concentration
BOD	mg/L	518 ± 5	12 ± 5
COD	mg/L	1350 ± 25	150 ± 5
SS	mg/L	1430 ± 12	140 ± 5
Turbidity	mg/L	464 ± 8	32 ± 5

**Table 3:** Freundlich and Langmuir isotherm model evaluation for the removal of BOD, COD, turbidity, and SS from PORE using tannin as a bio-coagulant.

Adsorbate	Freundlich Model			Langmuir Model		
	R^2^	K_f_ (L/mg)	*n*	R^2^	a (L/mg)	b (mg/mg)
BOD	0.9486	0.7705	2.6882	0.9371	0.0624	4.7778
COD	0.9375	0.0027	0.6515	0.8584	0.0016	18.116
Turbidity	0.9212	0.5937	2.9762	0.8768	0.0295	3.9825
SS	0.9193	0.4181	1.8352	0.8458	0.0035	18.7617

**Table 4:** Kinetics parameters for the removal of BOD, COD, turbidity, and SS from PORE using tannin as a bio-coagulant.

Parameters	T (°C)	*q_e_* (exp) (mg/mg)	Pseudo-First-Order Kinetics			Pseudo-Second-Order Kinetics		
			*q_e_* (mg/mg)	*k*_1_ (min^−1^)	R^2^	*q*_e_ (mg/mg min)	*K*_2_ (mg/mg min)	R^2^
BOD	30	2.54	3.06	0.0792	0.9548	2.88	0.0340	0.9951
	40	2.34	2.85	0.0654	0.994	2.76	0.0263	0.9954
	50	2.23	4.16	0.0707	0.9741	2.58	0.0236	0.9964
	60	2.21	5.95	0.0719	0.8729	2.24	0.0246	0.9991
COD	30	6.09	8.33	0.0772	0.9423	6.89	0.0140	0.9981
	40	5.79	6.04	0.0548	0.9498	5.95	0.0125	0.9973
	50	5.62	14.88	0.0730	0.9289	5.83	0.0093	0.9941
	60	5.58	8.78	0.0534	0.9695	5.34	0.0095	0.9891
SS	30	6.49	11.83	0.0633	0.8976	7.88	0.0068	0.9992
	40	6.23	13.59	0.0666	0.9594	7.52	0.0056	0.9921
	50	6.11	10.12	0.0511	0.9485	6.47	0.0056	0.9845
	60	6.05	10.08	0.0456	0.9361	6.22	0.0051	0.9799
Turbidity	30	2.18	3.86	0.0709	0.9238	2.75	0.0134	0.9699
	40	2.02	4.14	0.0652	0.9733	2.54	0.0203	0.9838
	50	2.11	4.14	0.0638	0.9559	2.51	0.0151	0.9637
	60	1.97	5.36	0.0696	0.9279	2.16	0.0177	0.9786

**Table 5:** Thermodynamics parameters for the removal of COD, BOD, turbidity, and SS from PORE using tannin as a polymeric coagulant.

T (K)	Δ*G*^0^ (kJ/mol)				Δ*H*^0^ (kJ/mol)				ΔS^0^ (kJ/mol)			
	COD	BOD	SS	Turbidity	COD	BOD	SS	Turbidity	COD	BOD	SS	Turbidity
303.15	−45.332	−83.505	−39.472	−82.544	−18.690	−39.267	−15.838	−38.003	0.088	0.146	0.078	0.147
313.15	−46.212	−84.965	−40.252	−84.014								
333.15	−47.091	−86.425	−41.032	−85.484								
353.15	−47.970	−87.885	−41.812	−86.954								
